# Supplementary material for: Predicting visual function by interpreting a neuronal wiring diagram
Source: Nature. 2024 Oct 2;634(8032):113–23. doi: 10.1038/s41586-024-07953-5 (PMC11446822; doi:10.1038/s41586-024-07953-5)
Supplement: Supplementary file 6 — CRF and ERF predictions for individual TmY4 and TmY9 cells. Analogous to Supplementary Data 3, but for TmY target types. Shown are the top four monosynaptic pathways, the strongest pathway passing through each of the top ten intermediary types (ranking from Extended Data Fig. 7), and the trisynaptic pathway Tm1–TmY–Dm3–TmY (see the section entitled Prediction of spatial normalization). [file 41586_2024_7953_MOESM6_ESM.zip › DataS4/TmY9q/720575940626152476.pdf]

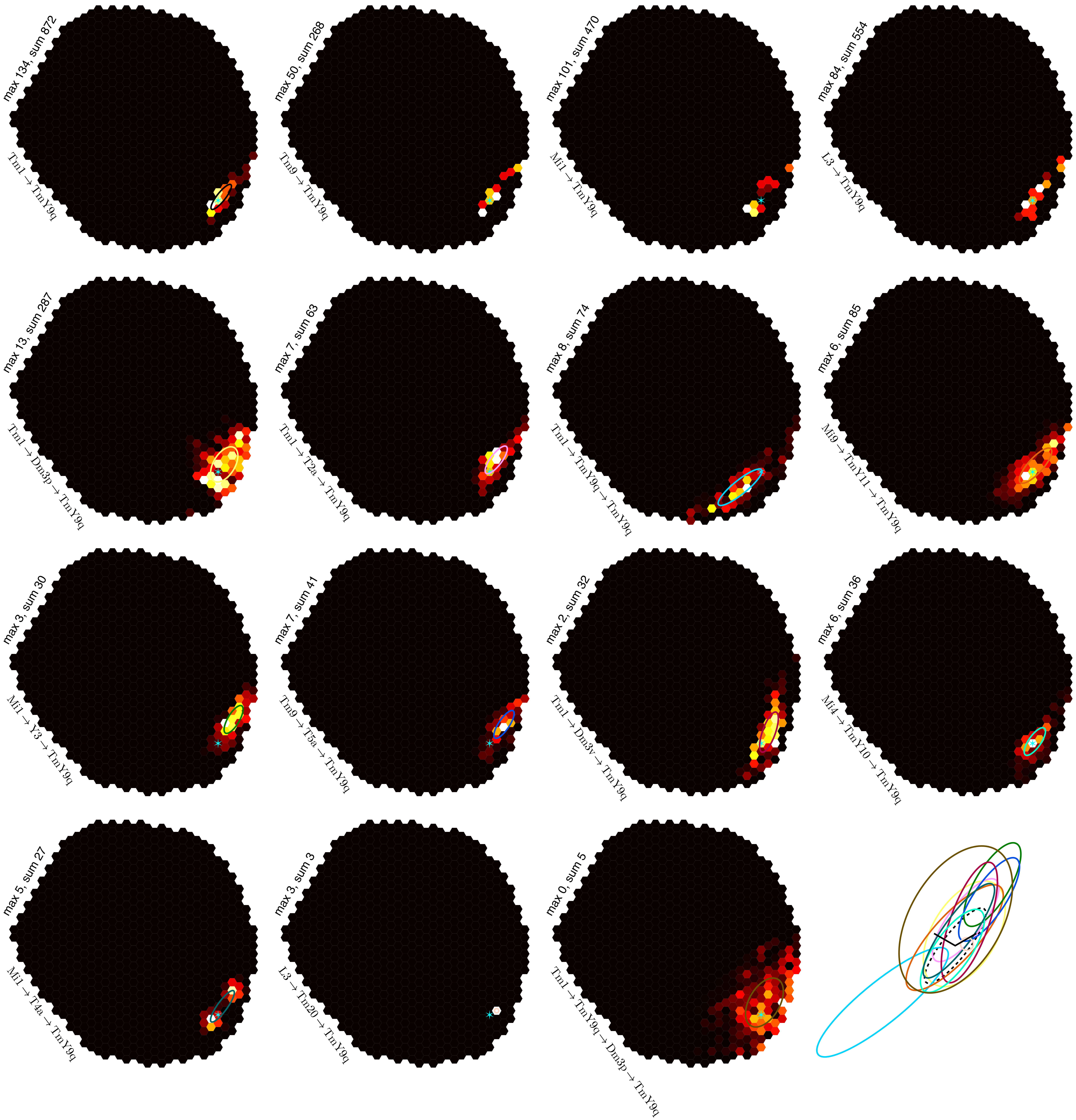

max 134, sum 872
Tm1 → TmY9q
max 50, sum 268
Tm9 → TmY9q
max 101, sum 470
Mi1 → TmY9q
max 84, sum 554
L3 → TmY9q
max 13, sum 287
Tm1 → Dm3p → TmY9q
max 7, sum 63
Tm1 → T2a → TmY9q
max 8, sum 74
Tm1 → TmY9q → TmY9q
max 6, sum 85
Mi9 → TmY11 → TmY9q
max 3, sum 30
Mi1 → Y3 → TmY9q
max 7, sum 41
Tm9 → T5a → TmY9q
max 2, sum 32
Tm1 → Dm3v → TmY9q
max 6, sum 36
Mi4 → TmY10 → TmY9q
max 5, sum 27
Mi1 → T4a → TmY9q
max 3, sum 3
L3 → Tm20 → TmY9q
max 0, sum 5
Tm1 → TmY9q → Dm3p → TmY9q
